# Prävention bei geriatrischen Patientinnen und Patienten in der Allgemeinmedizin

**DOI:** 10.1007/s00391-024-02358-5

**Published:** 2024-09-13

**Authors:** Reingard Glehr

**Affiliations:** Institut für Allgemeinmedizin und evidenzbasierte Versorgungsforschung, Medizinische Universität Graz, Michaeligasse 12, 8230 Hartberg, Österreich

**Keywords:** Kognitive Dysfunktion, Geriatrisches Assessment, Strukturierte Versorgung, Training, Biopsychosoziales Modell, Cognitive dysfunction, Geriatric assessment, Structured care, Exercise, Biopsychosocial model

## Abstract

**Hintergrund:**

Funktionsdefizite frühzeitig zu erkennen und diesen durch ein multimodales Behandlungskonzept gegenzusteuern, gehört zu den wichtigsten Aufgaben von Allgemeinmedizinerinnen und Allgemeinmedizinern, die meist primäre medizinische Ansprechpartner geriatrischer Patientinnen und Patienten sind.

**Ziel der Arbeit:**

Erläuterung von Strategien zur biopsychosozialen Begutachtung geriatrischer Patientinnen und Patienten sowie zur Erstellung individuell angepasster Präventionskonzepte in der allgemeinmedizinischen Praxis.

**Material und Methoden:**

Literaturrecherche zum theoretischen Hintergrund der wichtigsten Präventionsansätze bei geriatrischen Patientinnen und Patienten sowie Überlegungen zu Bedeutung und Umsetzung in der täglichen Praxis.

**Ergebnisse:**

Bei geriatrischen Patientinnen und Patienten sollten Präventionsmaßnahmen auf allen 4 Präventionsebenen simultan gesetzt werden. Die Förderung von körperlicher und geistiger Bewegung gilt als Schlüsselfaktor. Die Risiken Immobilität, Depression, kognitiver Abbau, Mangelernährung und nicht zuletzt Multimedikation haben besonderen Stellenwert.

**Diskussion:**

Geriatrische Patientinnen und Patienten stellen eine sehr heterogene Gruppe dar. Um individuell präventiv handeln zu können, braucht es eine multidimensionale Erhebung von Schlüsselfaktoren zum Erhalt von Funktionalität und relativer Gesundheit, trotz evtl. bereits bestehender Erkrankungen.

## Abstract (en)

**Background:**

Recognizing functional deficits early and counteracting them with a multimodal treatment concept is one of the most important tasks of general practitioners, who are usually the primary medical contact for geriatric patients.

**Aim:**

Illustration of strategies for a biopsychosocial assessment of geriatric patients and for the creation of individually adapted prevention concepts in general practice.

**Material and methods:**

Literature research on the theoretical background of the most important prevention approaches for geriatric patients as well as considerations on their relevance and implementation in daily practice.

**Results:**

For geriatric patients prevention measures should be implemented simultaneously on all four prevention levels. The main objective is promoting physical and mental exercise. The risks of immobility, depression, cognitive decline, malnutrition and, last but not least, polypharmacy are of particular importance.

**Conclusion:**

Geriatric patients represent a very heterogeneous group. In order to be able to take individual preventive action, a multidimensional assessment of key factors for maintaining functionality and relative health is required, even though chronic conditions may already exist.

Allgemeinmedizinerinnen und Allgemeinmediziner sind bei geriatrischen Patientinnen und Patienten oft primäre Ansprechpartner in Gesundheitsfragen und spielen gerade bei Früherkennung und Prävention eine zentrale Rolle. Funktionsdefizite frühzeitig zu erkennen und ihnen durch ein multimodales Behandlungskonzept gegenzusteuern, gehört zu ihren wichtigsten Aufgaben. Damit kann zum Erhalt der Lebensqualität von Patientinnen und Patienten wesentlich beigetragen und der Verlust von Unabhängigkeit hinausgezögert bzw. verhindert werden.

## Primärversorgung geriatrischer Patientinnen und Patienten 

Wird von geriatrischen Patientinnen und Patienten gesprochen, ist eine heterogene Gruppe gemeint. Diese setzt sich zusammen aus:

- gesund und aktiv alt Gewordenen,
- an einer chronischen Erkrankung Leidenden, jedoch gute Lebensqualität Besitzenden,
- mit Erkrankung pflegebedürftig Gewordenen [1].

In der allgemeinmedizinischen Praxis werden Patientinnen und Patienten aus allen Gruppen mit unterschiedlichsten Behandlungsaufträgen vorstellig. Der präventive Behandlungsansatz sollte in allen Gruppen im Fokus stehen. Verlangsamung von Abbauprozessen sowie Erhalt und Optimierung von Funktionsfähigkeit, Selbstständigkeit und Gesundheit sind die Ziele.

Oft werden Patientinnen und Patienten mit unspezifischen Symptomen, die frühen Erkrankungsstadien angehören oder auch nur Schwankungen der Befindlichkeit ohne Krankheitswert darstellen können, vorstellig. Die Arzt-Patient-Beziehung entwickelt sich in der Mehrzahl der Fälle zu einer Langzeitbeziehung, in der alle Ebenen des biopsychosozialen Modells beachtet werden sollten, in engem Austausch mit Kolleginnen und Kollegen der spezialisierten Medizin und nichtärztlichen Gesundheitsberufen [2, 3]. Neben kurativen Handlungen liegt der hausärztliche Fokus auf Gesundheitsförderung, Krankheitsvorbeugung sowie Empowerment der Patientinnen und Patienten. Letzteres bedeutet v. a. Unterstützung von Gesundheitskompetenz und Selbstmanagement.

Werden die Besonderheiten geriatrischer Patientinnen und Patienten und ihrer Versorgung (Tab. 1) betrachtet, prädisponiert dieser Zugang Hausärztinnen und Hausärzte dafür, wesentlicher Teil des Betreuungsnetzwerkes zu sein.

**Tab. 1:** Merkmale geriatrischer Patientinnen und Patienten und geriatrischer Versorgung. (Nach Schwartz [31])

Merkmale geriatrischer Patientinnen und Patienten	Hohe Prävalenz chronischer Erkrankungen und häufige Multimorbidität
	Hohe Prävalenz von Funktionsbeeinträchtigungen
	Verminderte Belastbarkeit und Adaptabilität
Ziele geriatrischer Versorgung	Besserung akuter Erkrankungen
	Linderung der Symptome chronischer Erkrankungen und Verhinderung einer Verschlechterung
	Erhalt der Autonomie
	Aufrechterhaltung sozialer Integration
Merkmale geriatrischer Versorgung	Komplexe Problemlagen, komplizierte Bewältigung
	Krankheit bedeutet nicht immer Versorgungsbedarf
	Betreuungsbedarf abhängig von der Fähigkeit zur Bewältigung des alltäglichen Lebens
	Behandlung möglichst ambulant, wenn die strukturellen Voraussetzungen vorhanden → soziale und pflegerische Hilfen oder Versorgung durch Angehörige mit entsprechender professioneller Unterstützung
	Langzeitorientiertes proaktives Management
	→ Versorgungskontinuität
	→ Krisenvorbeugung durch gute Kommunikation
	→ Rehabilitation

## Ebenen der Prävention

Der Unterschied zwischen präventiver und kurativer Medizin liegt im Fokus auf das Risiko – Prävention zielt einerseits auf eine Reduktion des Risikos, krank bzw. beeinträchtigt zu werden, andererseits auf die Reduktion eines zusätzlichen Risikos bei denen, die bereits eine Krankheit haben, ab. Risikofaktoren sollten abhängig von Alter, Konstitution, bereits bestehenden Erkrankungen sowie sich ergebenden Risikokonstellationen beurteilt werden.

Bei geriatrischen Patientinnen und Patienten sollten Präventionsmaßnahmen auf allen 4 Präventionsebenen simultan gesetzt werden (Tab. 2).

**Tab. 2:** Beispiele für Maßnahmen der 4 Präventionsebenen im Bereich der Geriatrie

Primärprävention	Regelmäßige Erhebung des Impfstatus bzw. Verabreichung saisonaler Impfungen
	Motivation zu Bewegung
	Motivation zu gesunder Ernährung
	Motivation zu intellektueller Tätigkeit sowie zur sozialen Interaktion
Sekundärprävention	*Strukturierte Früherkennung von Krankheiten*
	Karzinome von Mamma, Lunge, Cervix, Prostata, Kolon, Haut
	Bauchaortenaneurysma, Karotisstenose, Herzrhythmusstörungen (insbesondere Vorhofflimmerarrhythmie), COPD
	Endokrinologische Störungen, insbesondere Diabetes mellitus und Schilddrüsenerkrankungen
	*Erkennen von Risiken und Funktionsdefiziten*
	Bewegungsmangel
	Degeneration des Bewegungsapparates
	Schmerzen
	Rauchen
	Über‑, Mangel- oder Unterernährung
	Herz-Kreislauf-Risiken: Blutdruck, Blutzucker, Lipide
	Elektrolytentgleisungen
	*Achtsamkeit bezüglich mentaler Störungen*
	Depression: Beachtung von Hochrisikogruppen (Pflege Angehöriger, zerebrovaskuläre Erkrankung, Krebs, chronische Schmerzen, Immobilität)
	Demenz (geistige Aktivität, soziale Teilhabe)
	Essstörungen
	Alkoholproblematik
	Medikamentenabhängigkeit (Analgetika, Benzodiazepine u. a.)
	*Altersbedingte Veränderungen mit Chancen durch frühe Intervention*
	Osteoporose, Sarkopenie, Herzschwäche, Niereninsuffizienz
Tertiärprävention	Proaktive, strukturierte Versorgung chronischer Erkrankungen
	Regelmäßige Verordnung rehabilitativer Maßnahmen bei chronischen Erkrankungen
	Verordnung von Hilfsmitteln (Gehhilfen, Umbaumaßnahmen etc.)
	Stärkung des Selbstmanagements durch Steigerung der Gesundheitskompetenz
Quartärprävention	Bewahrung vor Überdiagnostik, z. B. Karzinomabklärung von Prostatahyperplasie bei Frailty-Patienten
	Bewahrung vor Übertherapie, z. B. invasives Management bei Frailty-Patientinnen und Patienten mit stabiler KHK
	Nutzen-Risiko-Abwägung, z. B. Antikoagulation bei rezidivierenden Stürzen

*COPD* „chronic obstructive pulmonary disease“, *KHK* „koronare Herzkrankheit“

## Assessment in der Hausarztpraxis

Um individuell präventiv handeln zu können, braucht es eine multidimensionale Erhebung von Schlüsselfaktoren zum Erhalt von Funktionalität und Gesundheit. Anlässe dafür können sein: Gesundheits- oder Vorsorgeuntersuchungen, Impfungen, Kontakt nach einem Krankenhausaufenthalt oder nach einem Sturz, Kontakt zu neuen Patientinnen und Patienten oder zu jenen, die nach längerem Zeitraum wieder in der Praxis vorstellig werden [4]. Ein geordnetes Assessment sollte helfen, Symptome zu objektivieren, heikle Themen anzusprechen und nach Maßnahmeneinleitung zur Verlaufskontrolle sowie zur Kommunikation mit anderen Berufsgruppen genutzt werden [5]. Erfolgt keine strukturierte Beurteilung, können Gleichsetzung von Alterserscheinungen mit Krankheit, schleichender Beginn von Einschränkungen und Vulnerabilität, Maskierung oder Bagatellisierung von Symptomen sowie ärztliche Angst vor Stigmatisierung ein Erkennen entscheidender Faktoren verhindern.

Mögliche Fragen zur Erfassung der biopsychosozialen Gesundheit einer geriatrischen Patientin/eines geriatrischen Patienten sind in Tab. 3 festgehalten. Aufgrund der zumeist begrenzten Zeit in der allgemeinmedizinischen Praxis ist diese ganzheitliche Begutachtung nicht in einer einzigen Konsultation durchführbar. Wiederkehrende Vor- und Einbestellungen der Patientinnen und Patienten ermöglichen in der Summe aber einen Gesamtüberblick.

**Tab. 3:** Beurteilung geriatrischer Patientinnen und Patienten in der Hausarztpraxis. (Nach Raetzo und Gaspoz [32], erweitert durch Huter [33])

Erfassung der biopsychosozialen Gesundheit einer geriatrischen Patientin/eines geriatrischen Patienten	
Funktionelle Begutachtung	Gang (Kleinschrittig? Unsicher? Schwankend? Notwendiges Hilfsmittel?)
	Überblick muskulärer Status, Feinmotorik, Tremor
	Fähigkeit, sich selbst anzuziehen
	Hygienestatus
Medikation	Sturzrisikofördernde Medikamente (Benzodiazepine, Psychopharmaka, Antihypertensiva, Diuretika …)
	Medikamentenplan? Medikamentenmanagement
	Adhärenz
	Potenziell gefährliche Dauermedikation (NSAR, Antikoagulanzien …)
Einschätzung der Sensorik	Sehvermögen
	Hörvermögen
	Polyneuropathie
Hautstatus	Offene Wunden
	Trockene Haut
	Pergamenthaut
	Hämatome
	Hauttumoren
Ernährungsstatus	Übergewicht
	Untergewicht oder Zeichen für Mangelernährung
	Zeichen für übermäßigen Alkoholkonsum
Kontinenz	Harn-/Stuhlkontinenz
Kognition	Zeitlich, örtlich, zur Person orientiert
Psychische Konstitution	Depressive oder ängstliche Grundstimmung
Soziale Umstände	Wohnsituation, finanzielle Situation
	Soziale Vernetzung
	Familiäre Situation
Osteoporoserisiko	Eigene und familiäre Frakturanamnese
	Mögliche sekundäre Osteoporose
Kardiovaskuläre Risikofaktoren	Eigene und familiäre Anamnese in Bezug auf kardiovaskuläre Erkrankungen
	Arterielle Hypertonie, erhöhte Blutfette, erhöhte Blutzuckerwerte
Risikofaktoren für Krebs	Eigene und familiäre Anamnese bezüglich Karzinomen
	B‑Symptomatik (Gewichtsverlust, Nachtschweiß, vermehrte Müdigkeit)
	Alkohol‑, Nikotinabusus
Impfanamnese	Bestehender Schutz gegen Tetanus, Pertussis, Diphtherie, Polio, FSME
	Impfungen gegen Pneumokokken, Herpes zoster
	Saisonale Impfungen gegen Influenza, COVID-19, RSV-Infektion

*FSME* Frühsommer-Meningoenzephalitis, *NSAR* nichtsteroidale Antirheumatika, *RSV* respiratorisches Synzytialvirus

Beispiele für präventive Maßnahmen, die sich aus dem strukturierten Assessment ergeben, sind:

- Förderung der Bewegung,
- diätetische Interventionen,
- Förderung sozialer Kontakte,
- kognitive Stimulation,
- Impfungen,
- weitere Diagnostik zur evtl. Bewahrung vor kardiovaskulären Ereignissen, Nierenfunktionsstörung, endokrinologischen Störungen, Anämie,
- Einleitung rehabilitativer Maßnahmen (Physiotherapie, Ergotherapie, Logopädie),
- apparative Unterstützung für Gehen, Sehen, Hören,
- professionelle Alltagshilfe.

Im Wissen um bestehende funktionelle Einschränkungen oder Gebrechlichkeit einer Patientin/eines Patienten sind klinische Entscheidungen fundierter zu treffen, Patientinnen und Patienten und deren Angehörige besser aufzuklären und Nutzen-Risiko-Abwägungen erleichtert. Das Streben nach Erhalt von Lebensqualität trotz gesundheitlicher Einbußen muss Grundlage der gemeinsamen Entscheidungen im Arzt-Patient-Gespräch sein.

## Schlüsselfaktoren und präventive Behandlungsansätze

### Bewegungsmangel

Wesentliche Voraussetzung für den Erhalt der Selbstständigkeit im Alter sind ausreichende Muskelkraft, Balance, Standfestigkeit, Beweglichkeit sowie Ausdauer und Gehfähigkeit. Neben einer verbesserten funktionalen Leistungsfähigkeit und möglichen Bewältigung der Alltagsanforderungen trägt gezieltes körperliches Training zu Muskel- und Knochengesundheit mit Sturz- und Frakturprävention bei [6]. Es hat positive Auswirkungen auf Schlaf, Psyche, kognitive Leistungsfähigkeit und Lebensqualität [7]. Auch bisher inaktive Patientinnen und Patienten können im Alter noch von regelmäßigem Training profitieren [8]. Umgekehrt verkürzt physische Inaktivität die Lebenserwartung und erhöht das Risiko vieler Erkrankungen wie beispielsweise Herzinsuffizienz, Diabetes mellitus Typ 2 sowie Brust- und Darmkrebs [9]. Auch die tägliche Gesamtaktivität mit Haushalts‑, Gartenarbeiten sowie Spaziergängen trägt zur Risikosenkung bei [10], Patientinnen und Patienten sollten aber über den Unterschied zwischen Alltagsbewegung und Mehrwert von zusätzlichem körperlichen Training aufgeklärt werden. Zweckorientierte Alltagsaktivitäten sollten so lange wie möglich eigenständig erfolgen, Hilfsmittel wie Lift oder Auto möglichst wenig genutzt werden. Ausdauertraining sollte 150–300 min/Woche mit moderater Intensität (mäßige Anstrengung z. B. bei schnellerem Gehen/Nordic Walking, Radfahren, Schwimmen, Ergometertraining oder Tanzen) oder 75–150 min/Woche mit höherer Intensität (z. B. Joggen, schnelles Radfahren oder schnelles Schwimmen) oder je 50 min mit moderater und 50 min mit höherer Intensität durchgeführt werden. Immobilen Patientinnen und Patienten sollte Sitzgymnastik empfohlen werden.

Zusätzlich sollte zumindest 2‑mal/Woche Krafttraining erfolgen. Kardiovaskuläre und erkrankungsassoziierte Risiken sollten vor vermehrtem Trainingsbeginn abgeklärt werden [11]. Eine Maximalbelastung sollte vermieden werden. Um Koordination und Gleichgewicht zu verbessern, wird Ganzkörpergymnastik mit propriozeptivem Training zumindest 3‑mal/Woche empfohlen [12].

### Immobilität

Ein physiologischer Abbau der Muskelmasse ist bei der älteren Bevölkerung unausweichlich. Kommt passagere oder dauerhafte Immobilität dazu, beträgt dieser Muskelverlust bis zu 10 % des Ausgangswerts/Woche, einhergehend mit einer bis zu 40 %igen Reduktion der muskulären Kraft [13]. Rommersbach und Wirth zeigten in einer Studie bei älteren hospitalisierten Patientinnen und Patienten, dass Immobilität den größten Risikofaktor für Muskelverlust während eines stationären Aufenthalts darstellt, wohingegen zu Alter, Ernährungsstatus und Inflammation kein kausaler Zusammenhang gefunden werden konnte [14].

Wesentlicher Präventionsansatz ist die Vermeidung der Hospitalisierung. Erreicht werden kann dies insbesondere durch konsequente Infektionsprophylaxe mit entsprechenden Impfungen sowie durch Schutz vor übertragbaren Erkrankungen durch Desinfektion und Abstandswahrung. Außerdem trägt die proaktive Versorgung chronischer Erkrankungen mit Vorbeugung von Dekompensationen bei.

### Depression

Eine/einer von 10 Patientinnen und Patienten, die in der Hausarztpraxis vorstellig werden, leidet an depressiver Symptomatik. Insbesondere jene mit chronischen Erkrankungen sind gefährdet, wobei körperliche (z. B. Atemnot, abnehmende Mobilität), seelische (z. B. Abhängigkeit, fehlende Zukunftsperspektive) und soziale Belastungen (Einsamkeit, Pflegeproblematik) eine Rolle spielen. Leichte depressive Verstimmungen sind häufig und nicht zwingend behandlungsbedürftig. Das aktive Ansprechen der Symptomatik vonseiten der Ärztin/des Arztes ist trotzdem wesentlich, um Ausprägung und Leidensdruck richtig einschätzen zu können.

Zeichen der Depression bei geriatrischen Patientinnen und Patienten sind Reizbarkeit, Aggressivität, aber auch Verlangsamung, Apathie, Hypochondrie und sozialer Rückzug. Bei schwerer Ausprägung kommen Wahnideen, zunehmende Streitsucht oder Misstrauen gegenüber Familie und Freunden hinzu.

Bei bestehender Frühsymptomatik eignet sich als Screening der 2‑Fragen-Test [15]:

Fühlten Sie sich im letzten Monat häufiger niedergeschlagen, traurig bedrückt oder hoffnungslos?

Hatten Sie im letzten Monat deutlich weniger Lust und Freude an Dingen, die Sie sonst gern tun?

Das Erkennen einer Depression ist aus mehreren Gründen wesentlich. Sie kann Ausdruck einer anderen Erkrankung sein oder Nebenwirkung einer Medikation. Außerdem beeinflusst sie Komorbiditäten durch abnehmende Adhärenz bezüglich Medikation, Selbstmanagement und Selbstfürsorge [16, 17]. Eine passive, manchmal latent aggressive Haltung der Patientinnen und Patienten gestaltet die Arzt-Patient-Beziehung oft herausfordernd. Die mit einer Depression einhergehenden Konzentrations- und Auffassungsstörungen sind oft verknüpft mit der Sorge, an einer beginnenden Demenz zu leiden. Die Abgrenzung ist schwierig und erfordert meist die Beiziehung fachärztlicher psychiatrischer Expertise. Depressive Patientinnen und Patienten sind in der Mehrzahl der Fälle nicht desorientiert, geben auf Nachfragen beispielsweise Datum und Uhrzeit richtig an. Der Leidensdruck ist zu spüren, während Patientinnen und Patienten mit Demenz ihre Beschwerden eher bagatellisieren oder dazu neigen, Defizite zu verstecken [18]. Abgegrenzt werden müssen auch akute Trauerreaktionen, die durch den vermehrten Verlust von nahestehenden Personen im Alter häufiger werden. Sie sind zeitlich begrenzt, können aber in eine protrahierte Trauerreaktion bzw. in eine Depression übergehen.

### Suizid

Die Suizidrate steigt in Österreich mit dem Alter an. Das Suizidrisiko ist in der Altersgruppe der 75- bis 79-Jährigen fast 2,5-mal, ab 85 Jahren über 5‑mal so hoch wie jenes der Durchschnittsbevölkerung [19].

Beispiele für außerordentlich belastende Begleiterscheinungen des „Altwerdens“, die das Suizidrisiko akut erhöhen, sind der Verlust vertrauter Menschen, Wechsel der Wohnform oder der Beginn einer schweren Erkrankung.

Präventiv wichtig sind die immer wieder zu stellenden Fragen nach Hoffnungslosigkeit, Schuldgefühlen, Suizidgedanken. Wesentlich ist bei schwerer Symptomatik bzw. bestehender Suizidalität eine Erweiterung des Hilfsnetzwerkes durch Beiziehen externer Spezialisten sowie von Beratungs- und Betreuungsstellen, ohne die Rolle als Vertrauensärztin oder -arzt abzugeben.

### Kognitive Dysfunktion

Die Prävalenz kognitiver Dysfunktion wird in der Bevölkerung über 65 Jahre mit 12–18 % und mit ca. 25 % bei über 80-Jährigen angegeben [20]. Noch höher wird sie bei älteren Patientinnen mit kardiovaskulären Erkrankungen angenommen (16–35 %, [21]). Atherosklerose und vaskuläre Risikofaktoren wie Bewegungsmangel, Rauchen, arterielle Hypertonie, Diabetes mellitus und Hyperlipidämie sind deutlich mit kognitiven Einbußen assoziiert [22]. Nicht zuletzt spielen Interaktionen und Nebenwirkungen bei Polypharmazie eine Rolle [23]. Frühzeitig sollten präventive Maßnahmen zum Erhalt der Kognition empfohlen werden. Das Wissen um den kognitiven Status einer Patientin/eines Patienten ermöglicht außerdem, Behandlungsoptionen so zu wählen, dass kognitive Fähigkeiten möglichst lange erhalten werden können. Nützliche Tools für die Objektivierung der kognitiven Funktion in der allgemeinmedizinischen Praxis sind der Mini-Cog Test – ein kurzer Test, bei dem die Fähigkeit getestet wird, sich an 3 Wörter zu erinnern und eine Uhr zu zeichnen [24], oder der Mini-Mental-Status-Test.

Häufige Komplikation kognitiven Abbaus ist das Delir, prädisponierende Faktoren sind auch hier Frailty-Syndrom und Polypharmazie [25]. Das Delir ist mit höherer Morbidität und Mortalität assoziiert, im Fall von Infektionskrankheiten wie z. B. COVID-19 kann es bei geriatrischen Patientinnen und Patienten die einzige klinische Präsentation der Erkrankung sein [26].

### Multimedikation

Im Bereich des Medikamentenmanagements kommt Hausärztinnen und Hausärzten nach wie vor eine Schlüsselrolle zu. Die physiologische Veränderung von Pharmakokinetik und -dynamik im Alter, veränderte Konstitution sowie die Abnahme von Leber- und Nierenfunktion erfordern eine regelmäßige Evaluation der Medikation gemäß Tab. 4. Sie sollte bei chronisch kranken Patientinnen und Patienten zumindest einmal jährlich sowie jedenfalls bei Hinweisen auf Einnahmeprobleme oder Verschlechterung des Allgemeinzustands durchgeführt werden [27].

**Tab. 4:** Evaluation der Medikamente bei Multimedikation. (Modifiziert nach DEGAM [27])

Indikation (Überversorgung/Unterversorgung)
Evidenz
Dosierung
Anwendungssicherheit (korrekte Einnahme?)
Anwendbarkeit
Medikamenteninteraktionen
Krankheitsinteraktionen (z. B. Leber‑, Niereninsuffizienz)
Doppelverordnungen
Therapiedauer, insbesondere bei Akutmedikation
Einnahmeplan und Dispensierung
Adhärenz

### Mangelernährung

Patienten mit einer Mangelernährung weisen eine verminderte Funktionalität und Lebensqualität auf, haben ein deutlich höheres Risiko, zu sterben oder schwere Erkrankungskomplikationen zu entwickeln, sowie eine längere Krankenhausverweildauer [28].

Risikofaktoren sind

- Probleme der Mundhöhle:
  - Entzündungen der Zähne, des Zahnfleisches oder der Mundschleimhaut,
  - Zahnprothesenprobleme.
- Schluckstörung:
  - neurologische Erkrankungen,
  - Mundtrockenheit, Trockenheit der Schleimhäute,
  - Sarkopenie.
- Appetitlosigkeit:
  - Depression, Demenz, Einsamkeit,
  - akute psychosoziale Krise,
  - Nebenwirkung von Medikamenten (Benzodiazepine, Antidepressiva, Chemotherapeutika, Analgetika …),
  - veränderter bzw. verminderter Geschmacks- oder Geruchssinn.
- Erkrankungen des Magen-Darm-Trakts.
- Chronische Erkrankungen wie Niereninsuffizienz, Herzinsuffizienz, „chronic obstructive pulmonary disease“ (COPD), onkologische Erkrankungen usw.
- Motorische Einschränkungen der Hände.

Der Verdacht auf einen Mangelernährungszustand sollte sich bei ungewolltem Gewichtsverlust, trockener Haut und trockenen Schleimhäuten, eingefallenen Wangenknochen, zunehmender muskulärer Schwäche, auffallender Antriebslosigkeit oder anhaltender Hypotonie stellen.

Zur Objektivierung helfen Screenings wie das Grazer Mangelernährungsscreening [29] oder das Malnutrition Universal Screening Tool (MUST) für Erwachsene [30].

Multiprofessionelle Interventionen sind nach der Diagnose wesentlich; eine umfassende Ab- und Aufklärung der Patientinnen und Patienten sind essenziell.

## Fazit für die Praxis

- Prävention bei geriatrischen Patientinnen und Patienten ist ein breites Aufgabengebiet, das über Vermeidung von Krankheiten und Unfällen hinausgeht.
- Die strukturierte Anwendung des biopsychosozialen Modells sollte mehr als ein Schlagwort sein; Prävention sollte auf allen Ebenen ansetzen.
- Wichtige Entscheidungsgrundlage für Diagnostik und Therapie sind wiederholte strukturierte Assessments, bei denen Probleme, die in der anlassbezogenen Konsultation evtl. nicht angesprochen oder übersehen werden, erkannt werden können.
- Durch Erkenntnisse aus Assessments und anlassbezogenen Konsultationen entwickelbare individuelle Präventionsstrategien sind notwendig, um geriatrischen Patientinnen und Patienten in ihrer Vielfalt, insbesondere aufgrund unterschiedlich erhaltener Funktionalität, gerecht werden zu können.
- Das Selbstmanagement der Patientinnen und Patienten sollte im Fokus stehen. Trotz multiprofessioneller Unterstützung sollte es möglichst lange aufrechterhalten werden.
